# Superior Medical Councillor D. V. Straub

**Published:** 1884-07

**Authors:** 


Superior Medical Councillor D. v. Straub died in Stuttgart on the 19th of September, 1883, where he was born on the 17th of January, 1820. He studied at the local veterinary school, and since 1842 has been a regimental veterinarian and assistant teacher at the school. In 1855 he was appointed Medical Councillor and Technical Veterinary Adviser to the Royal Medical "Collegium," a position which he held until 1881, when he voluntarily resigned. He published quite a number of articles in "Heving's Repertorium." He continued in active life to his last day, notwithstanding his sickness.—*Deutsche Zeitschrift für Thiermedicin*, 1883, *Part I.*

